# Are false positives in suicide classification models a risk group? Evidence for “true alarms” in a population-representative longitudinal study of Norwegian adolescents

**DOI:** 10.3389/fpsyg.2023.1216483

**Published:** 2023-09-15

**Authors:** E. F. Haghish, Bruno Laeng, Nikolai Czajkowski

**Affiliations:** ^1^Faculty of Social Sciences, Department of Psychology, University of Oslo, Oslo, Norway; ^2^Faculty of Humanities, RITMO Centre for Interdisciplinary Studies in Rhythm, Time and Motion, University of Oslo, Oslo, Norway; ^3^Division of Mental and Physical Health, Department of Mental Disorders, Norwegian Institute of Public Health, Oslo, Norway

**Keywords:** suicide attempt, classification error, suicide risk, adolescents, false positive

## Abstract

**Introduction:**

False positives in retrospective binary suicide attempt classification models are commonly attributed to sheer classification error. However, when machine learning suicide attempt classification models are trained with a multitude of psycho-socio-environmental factors and achieve high accuracy in suicide risk assessment, false positives may turn out to be at high risk of developing suicidal behavior or attempting suicide in the future. Thus, they may be better viewed as “true alarms,” relevant for a suicide prevention program. In this study, using large population-based longitudinal dataset, we examine three hypotheses: (1) false positives, compared to the true negatives, are at higher risk of suicide attempt in future, (2) the suicide attempts risk for the false positives increase as a function of increase in specificity threshold; and (3) as specificity increases, the severity of risk factors between false positives and true positives becomes more similar.

**Methods:**

Utilizing the Gradient Boosting algorithm, we used a sample of 11,369 Norwegian adolescents, assessed at two timepoints (1992 and 1994), to classify suicide attempters at the first time point. We then assessed the relative risk of suicide attempt at the second time point for false positives in comparison to true negatives, and in relation to the level of specificity.

**Results:**

We found that false positives were at significantly higher risk of attempting suicide compared to true negatives. When selecting a higher classification risk threshold by gradually increasing the specificity cutoff from 60% to 97.5%, the relative suicide attempt risk of the false positive group increased, ranging from minimum of 2.96 to 7.22 times. As the risk threshold increased, the severity of various mental health indicators became significantly more comparable between false positives and true positives.

**Conclusion:**

We argue that the performance evaluation of machine learning suicide classification models should take the clinical relevance into account, rather than focusing solely on classification error metrics. As shown here, the so-called false positives represent a truly at-risk group that should be included in suicide prevention programs. Hence, these findings should be taken into consideration when interpreting machine learning suicide classification models as well as planning future suicide prevention interventions for adolescents.

## Introduction

In recent years, the application of supervised machine learning methods has led to a considerable improvement in the accuracy of suicide attempt classification (Franklin et al., 2017; Walsh et al., 2017; Burke et al., 2020; Healy, 2021; Ley et al., 2022; Haghish et al., 2023). Although suicide attempts tend to have a low prevalence in population-representative samples, even a small detection error rate could result in a large number of misclassifications. In particular, a substantial portion of the classification error would constitute False Positives (FP, falsely labeled as suicidal) since most of the population is comprised of non-suicidal people. In recent research on machine learning suicide classification, FP are deemed irrelevant for intervention and are not considered to be at risk of attempting suicide. For example, Linthicum et al. (2019, p. 220) underscored that: “*In the case of false positives, individuals who are not at risk will be classified as being at risk*,” a point that is also emphasized in van Vuuren et al. (2021, p. 1418) paper: “*whether it is acceptable to label … [FP] as at risk, when they are actually not.”*

**Figure 1:**
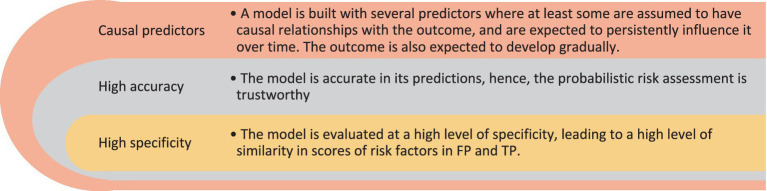
Necessary conditions to conceptualize FP as a risk group.

Recently, Haghish and Czajkowski (2023) proposed a theoretical explanation as to why, when numerous psycho-socio-environmental risk factors are incorporated into machine learning retrospective suicide attempt classification models, false positives may be at a higher risk of future suicidal behavior compared to true negatives. As summarized below in Figure 1, they considered three preconditions: (1) causal relationships between predictors and the binary outcome (i.e., suicide attempt) are expected to persistently influence the outcome over time; (2) high accuracy for the model ensuring that the estimated suicide attempt risk is accurate; and (3) a high level of specificity for the classification based on estimated probabilities. Based on these assumptions, Haghish and Czajkowski (2023) postulated that, for accurate models of suicide attempts classification that are trained with a multitude of risk factors and when the specificity threshold of the model is set to be high (i.e., a high cutoff value for classification is considered), it is likely that FP would be at high risk of attempting suicide in the future.

In the present study, we test this hypothesis using a comprehensive population-based longitudinal data from Norwegian adolescents. We develop a model for suicide attempt classification based on the first time point (T1) data and identify FP and TN. Next, we examine the prevalence with which FP and TN report suicide attempts for the first time within a 2-year frame at the second time point (T2) data and compare their respective suicide attempt risks. Specifically, we address three hypotheses: (1) the prevalence of suicide attempts at T2 will be notably higher among FP compared to TN; (2) within the FP group, adolescents with a higher risk score at T1 will more likely report a first-time suicide attempt at T2; (3) The reason we expect such a trend is that, as classification is made based on higher thresholds of estimated suicide attempts risk scores, the severity of known suicide attempt risk factors (e.g., depression, anxiety, non-suicidal self-harm, and suicide ideation) also increases among FP. Thus, an increase in similarity between FP and TP would account for the machine learning model classifying the FP as suicidal (Haghish and Czajkowski, 2023). Simply put, with higher cutoff values, the similarity of FP and TP groups on different risk factors will increase. This understanding of false positives renders the FP a group of interest, both from a methodological and clinical point of view. To our knowledge, this is the first research article to examine whether FP, in the context of machine-learning retrospective suicide classification constitutes a risk group and how this risk is influenced by the choice of specificity.

## Methods

### Sample

We analyze data from the *Young in Norway* study (https://ung-i-norge.no). In 1992, 11,369 adolescents (5,630 girls and 5,739 boys) from 67 schools in different municipalities participated in the study. A minority of participants did not respond to the suicide attempt item and were dropped from the analyses; i.e. 537 (4.72%, 307 boys and 230 girls). The remaining 10,832 participants ranged in age from 12 to 20 years (mean = 15.75, SD = 1.90). We refer to this sample as T1, by being collected in the first time point. The second wave of data collection was carried out 2 years later in 1994 and 8,018 participants responded to the questionnaire, of which, 593 participants (7.40%) did not answer the suicide attempt item and were excluded from the analyses. We refer to this sample as T2 throughout the article.

### Measures

The questionnaire contained items assessing the adolescents’ socio-demographic background such as family affluence and cultural capital (Bourdieu, 2018), personal development (e.g., puberty, sexual activities, physical disabilities, etc.), family learning environment (Marjoribanks, 1987), school environment, academic performance, and educational self-efficacy. In addition, adolescents’ attitude toward their future occupation was measured with occupational aspiration (Storvoll and Wichstrøm, 2002), career incentives (see Bores-Rangel et al., 1990), and career decision profile (Jones, 1989; Jones and Lohmann, 1998). Personality development was measured with several instruments including the *Bem Sex Role Inventory* (Bem, 1974; Lenney, 1991), a revised version of *extended objective measure of ego identity status* (Grotevant and Adams, 1984), Rosenberg’s *stability of self* (Alsaker and Olweus, 1986), *the self-perception profile for adolescents* (Wichstrøm, 1995), *state–trait Anger expression inventory* (Spielberger et al., 1999), *Barratt impulsiveness scale* (Patton et al., 1995), and Marlowe-Crown social desirability scale (Crowne and Marlowe, 1960). Finally, the questionnaire also included a variety of instruments assessing conduct, anxiety, and mood disorders. These mental-health related instruments were *Olweus’ scale of antisocial behavior* (Olweus, 1989), *substance use* (Pape and Rossow, 2004), the *Bulimic investigatory test* (Henderson and Freeman, 1987), *the eating attitude test* (Garner and Garfinkel, 1979), the *Cantril ladder scale* (Cantril, 1965), *the UCLA loneliness scale* (Russell et al., 1978, 1980), *Hopkins symptom checklist* (Derogatis et al., 1974), and *depressive mood inventory* (Kandel and Davies, 1982).

Participants also were asked to respond to the item “*Have you ever tried to take your own life*,” assessing attempted suicide, which could be answered in with a binary “yes” or “no.” This item was used as the outcome variable for the classification task. The same item was examined at T2, allowing us to identify participants who had not reported a suicide attempt at T1 and are reporting a suicide attempt at T2.

## Analysis

### Model training and model selection

Utilizing the Gradient Boosting Algorithm (GBM; Friedman, 2001), we trained a binary classification model to identify suicide attempts at T1. The dataset was randomly divided into training (70%) and testing (30%) subsets. We fine-tuned the GBM algorithm with random search on the training dataset and employed a 10-fold Cross-Validation (CV) method to assess the performance of the models. The search algorithm was optimized to select models with highest Area Under Precision-Recall Curve (AUPRC). This metric is considered less biased than Area Under the Curve (AUC) or misclassification rate, especially when outcomes are rare and severely imbalanced (Davis and Goadrich, 2006; Chicco, 2017). We chose the model showcasing the highest AUPRC and evaluated its performance on the testing subset. In addition to AUPRC, we also analyzed the Receiver Operating Characteristic (ROC) curve and reported the AUC of the chosen model to make our results comparable with the literature. Note that the procedure of model training and model selection is exclusively conducted on T1. As detailed below, T2 is only used as a follow-up to examine the risk of FP and TN classification groups.

### Classification based on different specificity thresholds

**Figure 2:**
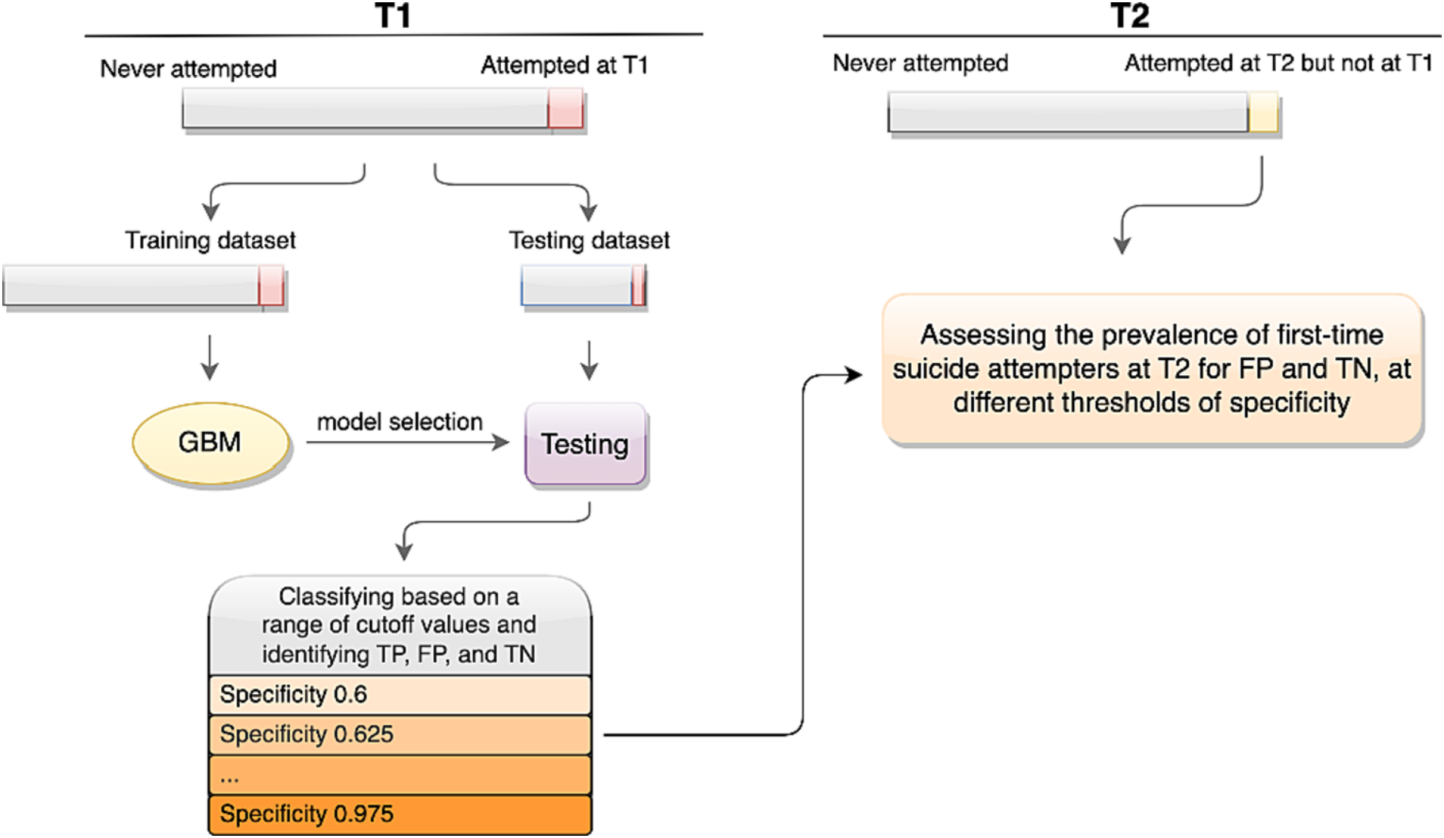
The study procedure.

The machine learning classification model assigns a risk score for each subject in the test dataset, which can range from 0 to 1. The higher the estimated suicide risk score, the higher the chance of a past suicide attempt. Classification can be performed based on any chosen threshold value in this range, resulting in different rates of FP, TN, and True Positives (TP). The higher the cutoff value for classification, the less likely it is that individuals are falsely classified as positive. This, however, comes at the cost of misclassifying a higher proportion of true positive individuals who were assigned a lower risk score, thus increasing the False Negative (FN) group. We used the adjROC R package (Haghish, 2022a) to perform the classification for cutoff values corresponding to specificity levels ranging from 0.60 to 0.975 and accordingly, for each level we identified TP, TN, and FP. Crucially, all classifications were made based on the T1 data of the testing dataset only. In other words, using the selected model and T1 test dataset, we classified the test sample for a range of cutoff values, gradually increasing the specificity of the classification model and subsequently, identified the individuals which would be classified as TP, FP, and FN at each specificity threshold. Specifically, the T2 dataset was only used to examine the prevalence of suicide attempts among these classification groups. The procedure of model training, model selection, classification for a range of specificity values, and estimating relative risk for the first-time attempters at T2 is shown in Figure 2.

Next, we assessed the ratios with which FP and TN reported a suicide attempt for the first time at T2. We calculated the Relative Risk ( $RR_{FP/TN}$ ) as shown below. Fisher’s exact test of count data was used to evaluate whether the relative risk values were significantly greater than 1.0. Finally, we calculated the $RR_{FP/TN}$ as a function of the level of specificity to examine whether the relative risk increases for higher values of estimated risk, corresponding to higher specificity cutoff for the model as well.


$$RR_{FP/TN}=\frac{\mathrm{suicide}\;\mathrm{prevalence}\;\mathrm{at}\;T2\;\mathrm{among}\;FP}{\mathrm{suicide}\;\mathrm{prevalence}\;\mathrm{at}\;T2\;\mathrm{among}\;TN}$$

### Missing data imputation

Missing observations in the predictors at the first time point were imputed using the mlim R package (Haghish, 2022b). The mlim package applies machine learning algorithms for missing data imputation and can handle mixed data types with complex interactions. This imputation algorithm is shown to result in lower imputation error compared to standard statistical procedures (Haghish, 2023). The outcome variable (i.e., suicide attempt) was separated from the dataset prior to the imputation and thus, missing data on the outcome variables were removed. After the imputation, the outcome variable was reattached to the dataset.

## Results

**Figure 3:**
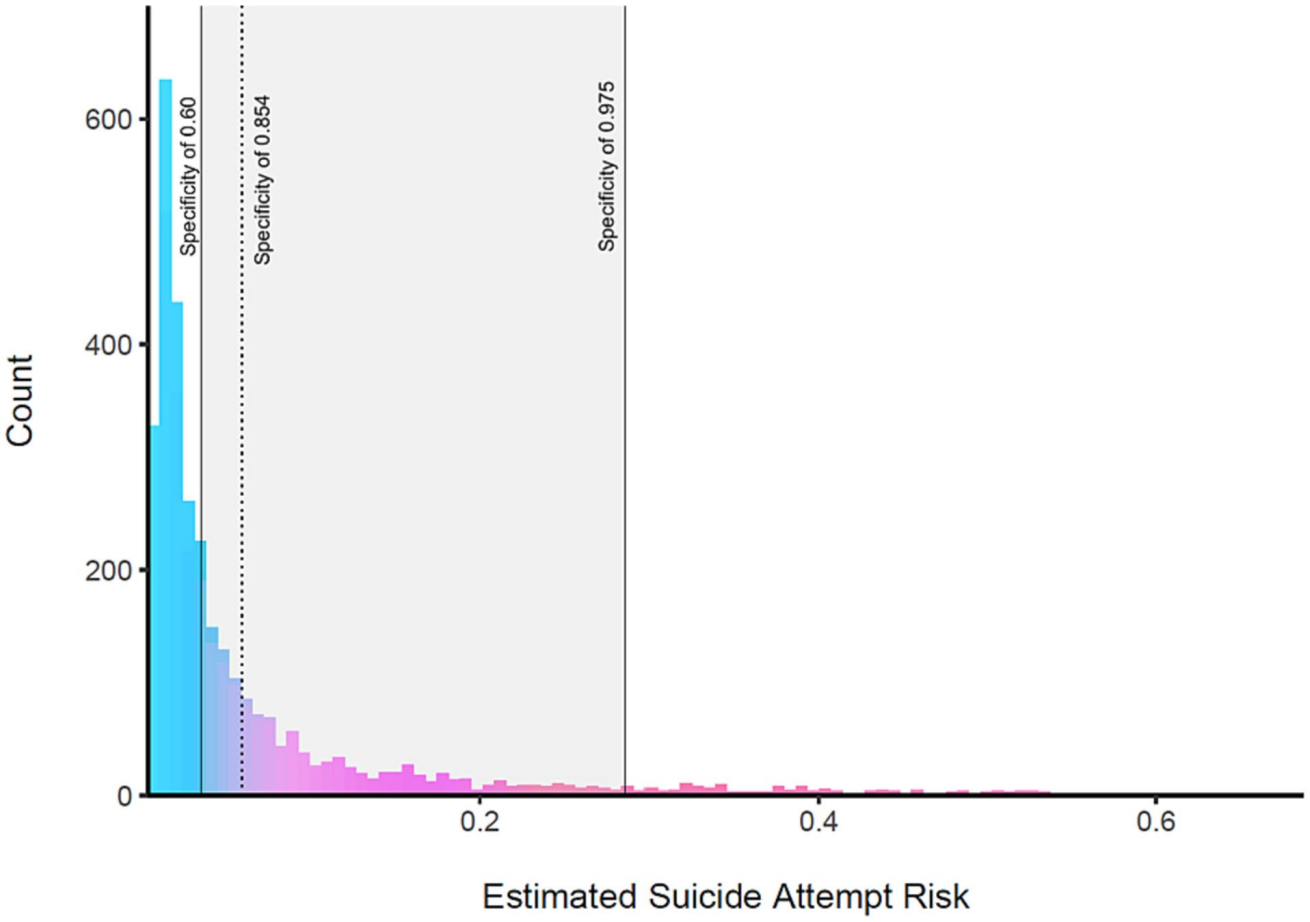
The estimated suicide attempt risk of the test dataset at T1. The gray rectangle indicates the range of estimated suicide attempt risk that corresponds to specificity levels ranging from 0.60 to 0.975, showing risk values ranging from 0.0346 to 0.286. As higher risk thresholds (values) for classification are selected, the specificity of the model also increases accordingly.

In the first time point, 7.52% of the items were missing and therefore were imputed prior to the analysis. A previous suicide attempt at T1 was reported by 8.43% (*n* = 913) of the adolescents, of which 37.79% (*n* = 345) were boys and 62.21% (*n* = 568) were girls. Of the reported suicide attempts, 57.61% were by adolescents in senior high school (above 15 years old) and the rest (42.39%) in junior high school. Fine-tuning the algorithms, the best GBM model reached AUPRC of 50.51% and AUC of 88.58%. Analysis of the model suggested a cutoff of 0.0587, resulting in sensitivity of 0.854 and specificity of 0.758. This cutoff value is shown in Figure 3 with a dotted line, alongside the histogram of the estimated suicide risk scores for the test dataset. Further analysis using the adjROC R package estimated that cutoff values ranging from 0.0346 to 0.286 would correspond to specificity values ranging from 0.60 to 0.975, respectively.

### False positives’ relative risk

**Figure 4:**
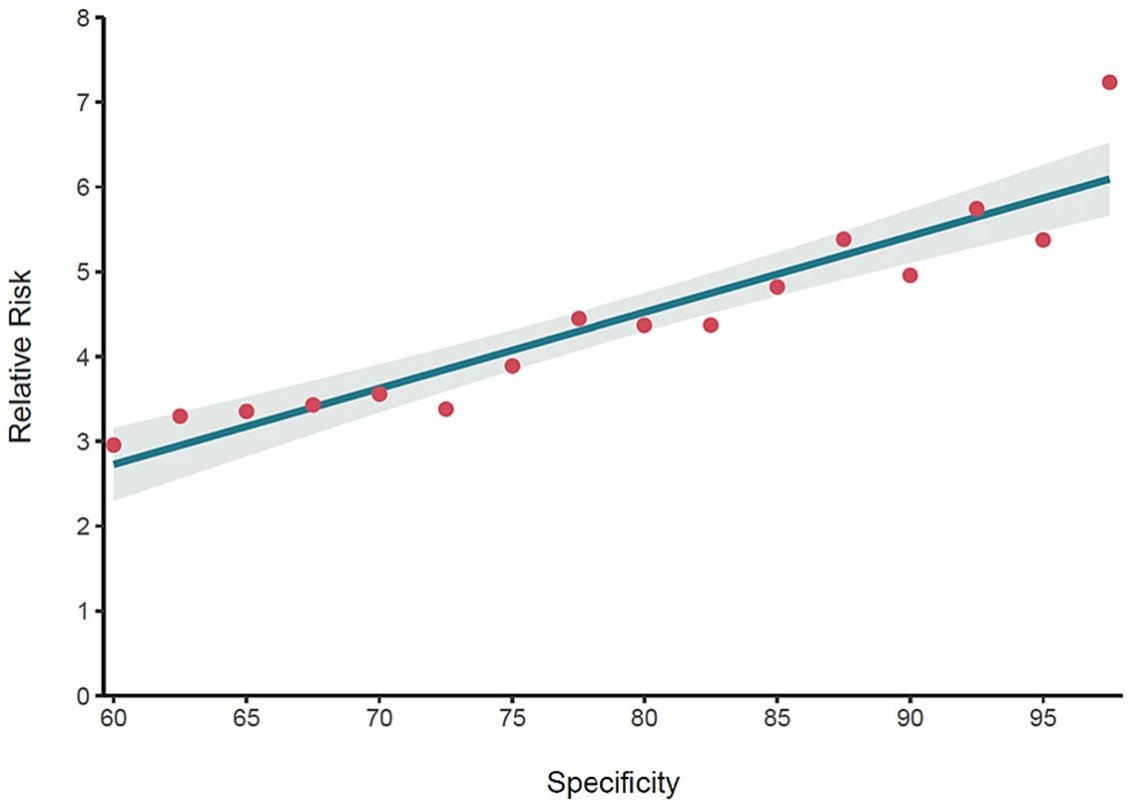
The relative risk of false positives at T2 for different specificity values. The red points represent the relative risk of the FP group at T2, indicating that FP are at much higher risk of attempting suicide compared to TN. The regression line indicates that the relative risk of the FP increases as a higher specificity is selected for the model.

At T2, 156 individuals reported their first suicide attempt. We determined which of these individuals were in the TN or FP groups based on different specificity thresholds. As depicted in Figure 4, we computed the $RR_{FP/TN}$ corresponding to rising specificity levels. This figure also displays the regression line and its 95% confidence interval for various specificity values. As shown in Figure 4, the relative risk spans from 2.96 for a specificity of 0.60 to 7.22 at a specificity of 0.975. Fisher’s test indicated that both risks are significantly higher than 1.0 (RR_specificity = 0.6_ = 2.96, 95% *CI* = 1.74 – Inf, *p* = 0.0002 and RR_specificity = 0.975_ = 7.22, *95% CI* = 3.06 – Inf, *p* = 0.0002). Moreover, as shown in Figure 4, the specificity threshold related to the FP’s relative risk significantly increased with increasing specificity [*Adjusted R^2^* = 0.865, *F* (1, 14) = 96.750, *p* < 0.0001].

### Similarity of false positives and true positives

**Figure 5:**
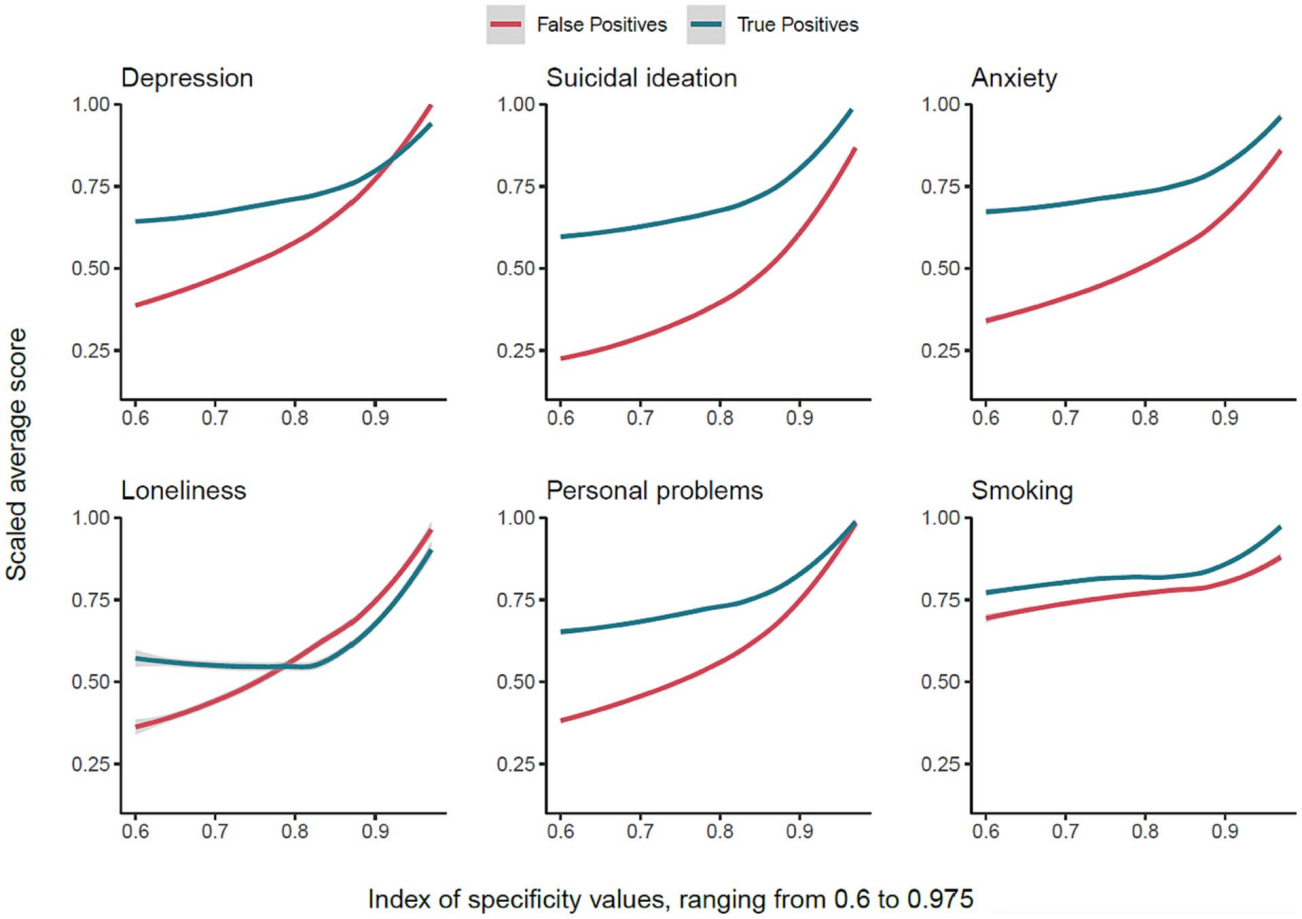
The mean score of FP increases alongside specificity and becomes similar to TP.

In Figure 5, we plotted the normalized average severity of symptoms of depression, suicidal ideation, general anxiety, perceived loneliness, perceived personal problems (evaluated with an item asking “*do you have a personal problem that you need help with*”), and frequency of smoking among FP and TP for specificity levels ranging from 0.60 to 0.975. Apart from smoking, the average scores for the other variables in the FP and TP groups were more similar for higher levels of specificity. This was especially pronounced when specificity was above 0.9. In Figure 5, for both TP and FP, smoking is frequent. However, for high-risk adolescents, where specificity was above 0.9, smoking behavior appeared to be even more frequent.

To examine the third hypothesis, we modeled the difference between the two curves for each scale, which generally resulted in a linear trend, diminishing with increasing specificity. Apart from the smoking frequency that had a negligible reverse trend from specificity of 0.9–0.975 [a*djusted R^2^* = −0.016, *F* (1, 36) = 0.42, *p* = 0.52], other symptoms conformed to the anticipated trend, such as depression [a*djusted R^2^* = 0.969, *F* (1, 36) = 1163.0, *p* < 0.0001], suicide ideation [a*djusted R^2^* = 0.963, *F* (1, 36) = 974.2, *p* < 0.0001], anxiety [a*djusted R^2^* = 0.959, *F* (1, 36) = 870.6, *p* < 0.0001], loneliness [a*djusted R^2^* = 0.797, *F* (1, 36) = 146.0, *p* < 0.0001], and personal problems [*adjusted R^2^* = 0.954, *F* (1, 36) = 751.0, *p* < 0.0001].

## Discussion

Within the context of machine-learning-based suicide attempt classification, we investigated three hypotheses concerning the suicide attempt risk of the FP group. Assuming the classification is executed by a highly precise model, our first hypothesis posited that, in comparison to the TN group, the FP group would exhibit a substantially elevated risk of suicide attempts over a two-year span. The fine-tuned model reached AUC of 88.58%, meeting the precondition that the classification model is highly accurate (Šimundić, 2009). The results supported this hypothesis. In the two years after the initial assessment, the FP group’s self-reported suicide attempts’ prevalence was approximately 3 to 7 times that of the TN group. This statistically significant rise in relative risk indicates that a notable portion of the FP group may contemplate suicide in the future, particularly if the classification is made at a high specificity threshold. The second hypothesis suggested that the relative risk for the FP group would increase when classifications are set at higher specificity thresholds. The linear regression analysis showed a significant linear trend in support of this hypothesis. Finally, the third hypothesis proposed that as classification is made at higher specificity thresholds, the severity of the FP group’s suicide attempt risk factors would more closely resemble those of the TP group. Apart from frequency of smoking, a clear trend was observed for other mental health indicators such as depression, anxiety, suicidal ideation, loneliness and personal problems, which supported this hypothesis. Overall, these findings are in line with Haghish and Czajkowski (2023) argument that the FP group can be conceptualized as a suicide risk group and a relevant target group for a suicide intervention program rather than as a mere classification error.

There are several reasons why the FP group exhibited an elevated risk of suicide attempt in our study. First, our machine learning model was based on a large number of psychological, sociological, and environmental risk factors that are likely to be persistent and might have a causal influence on the development of suicidal behavior (Lohner and Konrad, 2006; Greening et al., 2008; Darke et al., 2010; Toprak et al., 2011; Lewis et al., 2014; Carballo et al., 2020; Haghish, 2023). Depression, anxiety, substance use, suicidal ideation, and other mental health risk factors are likely to endure over time (Caspi and Moffitt, 2018), keeping the FP at a higher risk of suicide attempt in the future. We showed that this effect would increase as a function of specificity. Moreover, as the model becomes more accurate in identifying TP and TN, the risk for those identified as FP also increases; presumably, because they were expected to have more similar patterns or levels on the risk indicators as the TP. However, we did not examine the above question, which should be addressed by future studies and, ideally, with even larger datasets. This type of effect can also be observed in logistic regression models, which utilize fewer predictors and assume monotonic relationships between the predictors and the outcome variable. In contrast, machine learning models do not assume such relationships and search for patterns in predictors, as well as interactions between them, in order to improve classification accuracy. Therefore, by increasing the specificity one can also assume that FP will have similar patterns to TP in their responses.

## Methodological and clinical implications

We showed that false positives - in the context of a machine learning retrospective suicide attempts classification model - can have a different interpretation than that usually ascribed. This finding is noteworthy from a methodological as well as a clinical perspective. From a methodological perspective, our results suggest that we might need a fairer method to evaluate machine learning model performance whenever FP are expected to be at high risk of developing the outcome. In short, when such a model is used in mental health settings, rather than punishing the model for its FP error, it should be credited for identifying individuals at risk, as long as such individuals are clinically relevant. This would seem a fairer and more optimistic way to evaluate the model performance rather than pessimistically consider all FP as sheer errors. In addition to common model performance procedures that are centered on misclassified groups, if the identified FP are within the conditions listed in Figure 1, the model evaluation could also be done based on clinical relevance. Such an approach clearly requires research to define “clinical relevance” for different health problems as well as estimating the relative risk of FP in different contexts rather than solely underscoring a correct classification. In the context of a hypothetical intervention and when the model’s accuracy is high and the classification is based on a high specificity, the individuals labeled as positive (whether TP or FP) are likely to benefit from the intervention. In this case, identifying individuals at high risk of becoming suicidal is clinically relevant for a prevention program.

In addition to severe symptoms of mental health problems, there are two other reasons why FP might be a relevant target group for a suicide prevention program. On the one hand, offering aid to individuals that already have attempted suicide does not guarantee a successful treatment, since there is little evidence in favor of effectiveness of suicide intervention programs for clinical samples (Large, 2018; Fox et al., 2020). Instead, preventing the development of suicidal behavior has been emphasized in recent studies as a better solution to reducing suicide prevalence in the population (Carter and Spittal, 2018; Haghish and Czajkowski, 2023). On the other hand, empirical evidence shows that even in Western countries such as Finland, Norway, and United States, most of adolescents’ suicide attempts might go undetected. Thus, they might not receive the needed professional mental health support before or after attempting suicides (Suominen et al., 2004; Olfson et al., 2012; Haghish, 2023). In the United States, for instance, college counseling centers have reported that only 19% of the students who died of suicide have been in contact with the counseling centers that are instructed to provide suicide first-aid (Gallagher, 2009). Therefore, identifying adolescents who are at high risk of becoming suicidal in future might be an indispensable step toward suicide prevention. Toward this end, machine learning can provide reliable suicide risk estimations, which can help us identify risk groups that need attention. As shown in our results, such estimated risk scores are indicative of future suicide attempt risk, even when the machine learning model is trained with retrospective data.

## Limitations and strengths

This study has several limitations that warrant attention. Primarily, suicide attempts were measured using a sole self-report item, leaving questions about the intensity and sincerity of these attempts. Nevertheless, this limitation does not undermine our conclusions which highlight that adolescents, even if inaccurately labeled as positive by a precise model, are at heightened risk of attempting suicide in the near future. Should the model incorporate more nuanced features reflecting the severity of suicide attempts, the relative risk associated with false positives is expected to escalate due to refined accuracy in risk estimation. In other words, the higher the model’s accuracy, the more reliable its risk predictions, irrespective of its classification correctness. Moreover, a binary classification is useful for identifying *who should receive help* and not *when the individual may attempt suicide*. Nonetheless, as previously mentioned, this method can play a pivotal role in prevention. By recognizing adolescents who are on the verge of developing suicidal tendencies, timely interventions can be administered, potentially averting tragic outcomes. Our study has also several notable strengths. Firstly, it takes a critical perspective on the common practice of suicide attempt classification with machine learning, shedding light on its inherent limitations. Furthermore, our findings accentuate the clinical significance of the FP group under the aforementioned preconditions, which merits more attention from future research. Finally, this study leverages a large population-representative longitudinal data from Norwegian adolescents, which helped to train an accurate model.

## Conclusion

We posited that the focus of suicide attempt classification should expand beyond those who have already attempted suicide to also encompass those poised to exhibit suicidal tendencies in the future. Notably, our findings suggest that it’s plausible to pinpoint both groups, if a machine-learning model for classifying suicide attempts integrates a multitude of psycho-socio-environmental risk factors, and achieves commendable accuracy and specificity. In other words, the more reliable the estimated suicide attempt risk, the more the risk should be taken seriously, even for misclassified adolescents. Additionally, achieving this would necessitate estimations concerning the fraction of FP group likely to undertake a suicide attempt or exhibit suicidal tendencies, warranting further empirical research. Furthermore, we argued that supplementary performance metrics could be incorporated to consider the potential risk or clinical relevance of FP. Our results should be taken into account by future suicide intervention programs that intend to use survey data and machine learning classification algorithms to identify at-risk individuals. As this is the first study to examine the claim that FP can be conceptualized as a risk group, there is a clear need for investigating further whether these results are replicable and can be extended to machine learning classification or prediction models of other mental health outcomes.

## Data availability statement

The data analyzed in this study is subject to the following licenses/restrictions: data from Young in Norway study was analysed. Researchers can apply for access to the data via https://ung-i-norge.no/. Requests to access these datasets should be directed to https://ung-i-norge.no/.

## Ethics statement

The studies involving humans were approved by the Ethical committee at the department of psychology, university of Oslo. The studies were conducted in accordance with the local legislation and institutional requirements. Written informed consent for participation in this study was provided by the participants’ legal guardians/next of kin.

## Author contributions

EFH developed the idea, carried out the analysis, and wrote the draft. BL and NC revised the manuscript. All authors contributed to the article and approved the submitted version.

## Conflict of interest

The authors declare that the research was conducted in the absence of any commercial or financial relationships that could be construed as a potential conflict of interest.

## Publisher’s note

All claims expressed in this article are solely those of the authors and do not necessarily represent those of their affiliated organizations, or those of the publisher, the editors and the reviewers. Any product that may be evaluated in this article, or claim that may be made by its manufacturer, is not guaranteed or endorsed by the publisher.
